# Microvessel density as new prognostic marker after radiotherapy in rectal cancer

**DOI:** 10.1186/1471-2407-9-95

**Published:** 2009-03-26

**Authors:** Saulius Svagzdys, Vaiva Lesauskaite, Dainius Pavalkis, Irena Nedzelskienė, Darius Pranys, Algimantas Tamelis

**Affiliations:** 1 Unit of Coloproctology, Department of Surgery, Kaunas Medical University Clinics, Eiveniu 2, Kaunas, Lithuania; 2 Institute of Cardiology, Kaunas University of Medicine, Kaunas, Lithuania; 3 Clinic of Pathological Anatomy, Kaunas University of Medicine, Kaunas, Lithuania; 4 Clinic of Dental and Oral Pathology, Kaunas University of Medicine, Kaunas, Lithuania

## Abstract

**Background:**

The extent of angiogenesis is an important prognostic factor for colorectal carcinoma, however, there are few studies concerning changes in angiogenesis with radiotherapy (RTX). Our aim was to investigate changes in tumor angiogenesis influenced by radiotherapy to assess the prognostic value of angiogenesis the microvessel density (MVD) in overall survival after radiotherapy.

**Methods:**

Tumor specimens were taken from 101 patients resected for rectal cancer. The patients were divided into three groups according to the treatment they received before surgery (not treated, a short course, or long course of RTX). Tumor specimens were paraffin-embedded and immunohistochemistry was performed with primary antibody against CD-34 to count MVD.

**Results:**

MVD was significantly lower in the group of patients treated with a long course of RTX (p <0.025). The mean MVD for the long RTX group was 134.8; for the short RTX group – 192.5; and for those not treated with RTX – 193.0. There were no significant statistical correlations between MVD and age, sex, grade of tumor differentiation (G) and tumor size (T) in those untreated with RTX. In long RTX group we found a significant prognostic rate for MVD when the density cut off was near 130 with 92.3% sensitivity and 64.7% specificity. When the MVD was lower than a cut off of 130, the survival period significantly increased (p = 0.001), the mortality rate is significantly higher if the MVD is higher than 130 (microvessel/mm^2^) (1953.047; p = 0.002), if the histological grade is moderate/poor (127.407; p = 0.013), if the tumor is T3/T4 (111.618; p = 0.014), and if the patient is male (17.92; p = 0.034) adjusted by other variable in model.

**Conclusion:**

Our results show that a long course of radiotherapy significantly decreased angiogenesis in rectal cancer tissue. MVD was found to be a favourable marker for tumor behaviour during RTX and a predictor of overall survival after long course of RTX. Further investigations are now needed to determine the changes in angiogenesis during a shorter course of RTX.

## Background

Colorectal cancer (CRC) is the third most common cancer and the fourth most frequent cause of cancer deaths worldwide [1]. CRC is a disease of the elderly, with only 5% of cases recorded in those younger than 40 years of age [2]. CRC is commonly found to be rectal cancer (RC). The main prognostic factors in RC are the size of the tumor (T), lymph node metastases (N) and grade of tumor differentiation (G) [3]. These factors are important for clinical staging and treatment. Preoperative radiotherapy with total mesorectal excision surgery improves local control, but has no statistically-significant impact on overall survival [4]. An efficient adjuvant treatment is necessary to further reduce the development of distant metastases and to improve overall survival. By contrast, preoperative chemoradiotherapy in terms of local control compared with radiotherapy alone is now proven in locally advanced rectal cancer, but overall survival is not yet modified by preoperative chemotherapy [5]. However little is known about the effects of radiation on endothelial cell behavior within these human tumors after radiotherapy (RTX). It is conceivable that RTX could diminish both proliferation and function of these endothelial cells (EC).

Common prognostic factors do not fully predict individual clinical outcomes especially among patients with tumors of stage II and III. The response of clinically-identical tumors to the same treatment may be vastly different. Therefore, to improve clinical care and to give an optimal treatment, biological prognostic markers must be identified [6]. The relationship between tumor angiogenesis with the grade of tumor differentiation, its metastatic potential and prognosis for survival has been proven [1]. Formation of new blood vessels from the endothelium of the existing vasculature is essential in angiogenesis [7]. The angiogenic process depends upon the balance between many stimulatory and inhibitory factors. Pro-angiogenic factors, such as vascular endothelial growth factor (VEGF), bind to sites on endothelial cells that lead to their proliferation. Microvessel density (MVD) is a surrogate marker which expressly reflects tumor angiogenesis and has been examined as a potential prognostic marker in numerous tumors [8]. The aim of our study was to investigate the changes in tumor angiogenesis under the influence of different types of radiotherapy to assess the prognostic value of angiogenesis (MVD) in the overall survival after radiotherapy.

## Methods

### Patients and tumor specimens

In this retrospective study, we assessed paraffin-embedded specimens from tumors from 101 patients resected for rectal cancer from January 2000 to December 2003. This study included patients with resectable rectal carcinoma. The exclusion criteria were a synchronous tumor or tumors in another localisation in anamnesis, or emergency surgery. The age of the patients was 69.4 ± 0.9 (ranging from 36 to 87 years of age); for males, the mean age was 68.7 ± 1.1 (n = 60), for females, mean age was 70.4 ± 1.5 (n = 41) years of age. All patients had confirmed rectal adenocarcinomas by histopathology and were staged according to the 5th edition of the American Joint Committee on Cancer (AJCC) Staging Manual [9]. All of the patients underwent radical low anterior or abdominperineal rectum resections. The patients were then divided into three groups according to the radiotherapy they had received before surgery. The first group (n = 57) only underwent surgery (no RTX), the second (n = 16) had 25 Gy short course radiotherapy (short RTX) and surgery, the third group (n = 28) had 48 – 52 Gy long course radiotherapy (long RTX) and surgery. The no RTX and short RTX group patients had clearly resectable tumors and a nodal status according to endorectal ultrasound examination was N0. Young-age patients and those with higher risk of disease recurrence (tumor differentiation grade etc.) with T3-T4 received short RTX. The patients with pre-treatment nodal status N1 by endorectal ultrasound or with unresectable T4 rectal tumors composed the long RTX group. All patients were followed up 42.6 ± 1.9 month after the operation for rectal cancer. The Lithuanian Cancer Register supplied the date and cause of death of those who died during the follow-up period. According to this information, study subjects were grouped as to those who survived or failed to survive during follow-up.

### Radiotherapy

A SIEMENS "MEVATRON M" 15 MV linear accelerator was used. Three dimensional conformal radiotherapy was used for all patients based on a contrast CT scan of the pelvis. The four fields „box“ technique was used. For short RTX group patients, irradiation was given in a dose of 5 Gy five fractions within five days. Surgical resection was performed within a week after RTX. For long RTX group patients, irradiation was given in a dose of 50 Gy/5 weeks with 2 Gy fraction treating 5 days/week. Surgical resection was performed within six – eight weeks after RTX.

### Immunohistochemistry

Paraffin-embedded specimens were sectioned in 3 μm slices and mounted on glass slides. De-paraffinization and re-hydration was performed by a slide stainer Varistain Gemini (ThermoShendon). Then, sections were washed with distilled water and heated in TRIS/EDTA buffer (pH 9.0) for 8 minutes at 110°C in a Microwave Histoprocessor RHS-1 (Milestone, Microwave Laboratory Systems). Immunochistochemistry was performed by the Shandon Coverplate system. After blocking endogenous peroxidase activity, all slides were incubated in the primary antibody directed towards CD-34 for 1 hour at a dilution of 1:100 in Antibody Diluent (DakoCytomation), followed by sequential 30 minutes incubations with Advance™ HRP Link and Advance™ HRP Enzyme (DakoCytomation). The binding reaction was detected by the Liquid DAB+ Substrate Chromogen System (DakoCytomation). Finally, the sections were counterstained with Mayer's hematoxylin (J.T.Baker), and mounted using xylene-based mounting medium Consul-Mount™ (Shandon).

### Evaluation of MVD

**Figure 1:**
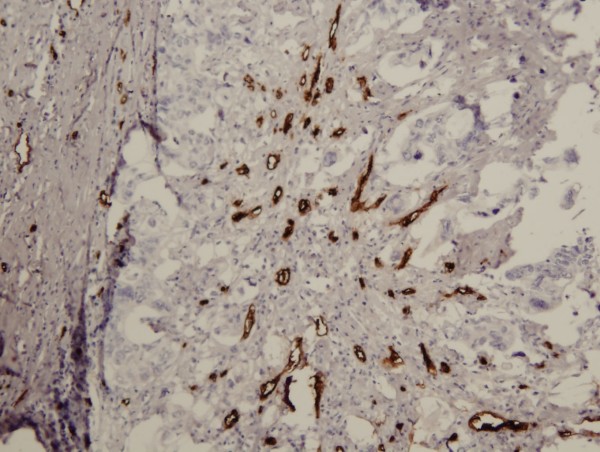
**The "hot spot" (original magnification ×10)**. MVD is 133 microvessel/mm^2^.

Slides stained with anti-CD34 monoclonal antibody were examined with an Olympus BX 61 light microscope. Single endothelial cells or clusters of endothelial cells positive for CD-34 were considered as a vessel. The presence of blood cells or fibrin without any detectable endothelial cells was not sufficient to define a microvessel. Vessels with muscular walls were not counted. At first, slides were examined at an original magnification of 4×. Three „hot spots“(areas with the highest microvessel concentration) from each slide were identified and these areas were photographed by a digital Olympus DP-70 camera at an original magnification of 10×. The area of this histological field was 576,207 μm^2^. Two independent observers with an Image – Pro AMS 6.0.0 program counted the number of microvessels in the histological field. MVD (microvessel/mm^2^) was then assessed according to Weidner et al. [10]. MVD of the specimen was estimated as a mean of MVD in three histological fields (Figure 1).

### Statistical analysis

For statistical analysis, SPSS version 13 (SPSS Inc., Chicago, IL) was used. The Students independent samples t-test, and ANOVA were used to compare means in groups. We used the Bonferoni test to compare multiple pairs. It was used as a precise or asymptomatic significance test due to the size of the samples. The ROC curves method was used to establish critical MVD value and later sensitivity and specificity of this parameter to predict the lethality issue. Survival curves were estimated by the Kaplan-Meier method and compared by the Mantel (log rank) test. Prognostic factors of survival were identified by the use of the Cox proportional hazard model. p < 0.05 was considered to be statistically significant.

### Ethical approval

The study was approved by Kaunas Regional Ethics Committee for Biomedical Research (protocol No.: 134/2006)

## Results

The main clinical data of the patients are summarized in Table 1. There was no statistically- significant difference between the groups according to age, sex, tumor T and death. The tumor T was higher and metastases in lymph nodes were more frequent in the group that had received no RTX as compared to the long RTX treatment group. This phenomenon was due to a radiotherapy downstaging effect.

**Table 1:** Clinical Data of Patient Groups Investigated

	Treatment Groups		
	
	No RTX (n = 57)	Short RTX (n = 16)	Long RTX (n = 28)
Age M ± SE, years	71.2 ± 1.2	66.0 ± 2.3	67.6 ± 1.6

Sex, %			
Male	45.6	43.8	28.6
Female	54.4	56.3	71.4

Tumor, %			
pT1/T2	22.8	37.5	35.7
pT3/T4	77.2	62.5	64.3

Lymph node metastases (pN), %			
Absent	50.9	56.3	75
Present	49.1	43.7	25

Histological grade (pG), %			
Very well/well	66.7	37.5	60.7
Moderate/poor	33.3	62.5	39.3

Mortality Rate, %	42.1	18.8	39.3

### Microvessel Density

**Figure 2:**
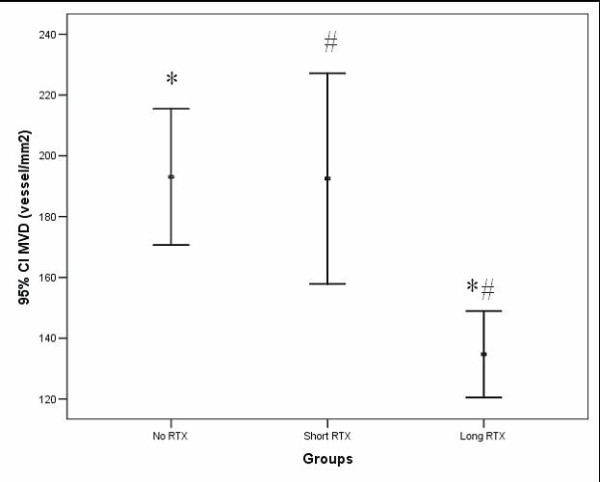
**Differences of mean ± SE of MVD (microvessel/mm2) in untreated (no RTX), short term (short RTX) and long term RTX (long RTX) groups**. F = 6.726; df = 2; p* = 0.002, p^# ^= 0.034.

The mean MVD in the no RTX, the short RTX and the long RTX groups was respectively 193.0 ± 11.2, 192.5 ± 16.2 and 134.8 ± 7.0 vessel/mm^2 ^(F = 6.726; df = 2; p = 0.002). The MVD was significantly lower in the samples from patients who had received the long course of RTX before operation (Figure 2).

### Long course RTX group

There was no significant difference in the MVD according to gender, T, N, and G in the long RTX group (Table 2).

**Table 2:** Microvessel density (MVD) in relation to gender, T, N and tumor differentiation grade in the long term RTX group.

	MVD M ± SE (microvessel/mm2)	p
Gender		

Male	131,46 ± 10.2	0,48

Female	136,07 ± 8.9	

Tumor		

pT1/T2	145,9 ± 14,2	0,38

pT3/T4	128,56 ± 7,4	

Lymph node metastases pN		

Absent	134.4 ± 8,8	0,48

Present	135.9 ± 10,1	

Histological grade pG		

Very well/well	134,4 ± 7,6	0,33

Moderate/poor	135,3 ± 13,7	

**Figure 3:**
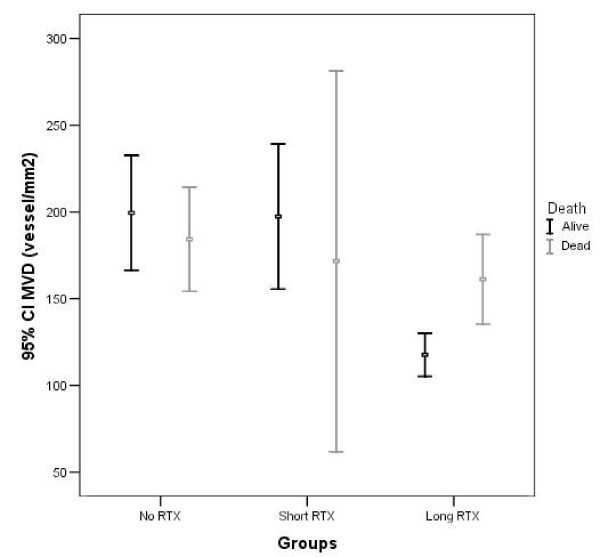
**Distribution between mean ± SE of MVD (microvessel/mm2) and survival in untreated (no RTX), short term (short RTX) and long term RTX (long RTX) groups**.

The patients who underwent long RTX and survived during the survey demonstrated significantly smaller MVD in tumor specimens and had lower lymph node metastatic rates in comparison to the patients who died during the survey. The mean MVD was 117.6 ± 5.8 vs. 161.2 ± 11.6 microvessel/mm^2^, (p = 0.001) and lymph node metastatic rate was 11.8% vs 45.5% (p < 0.05), respectively (Figure 3, Table 3).

**Table 3:** Clinical Data of the Patients who received a long course of radiotherapy

	Survived (n = 17)	Deceased (n = 11)	p
Age M ± SE, years	67.5 ± 1.9	67.8 ± 2.9	0.64

MVD M ± SE, microvessel/mm^2^	117.6 ± 5.8	161.2 ± 11.6	0.001

Sex, %			
Male	35.3	18.2	0.3
Female	64.7	81.8	

Tumor, %			
pT1/T2	41.2	27.3	0.45
pT3/T4	58.8	72.7	

Lymph node metastases (pN), %			
Absent	88.2	54.5	0.044
Present	11.8	45.5	

Histological grade (pG), %			
Very well/well	64.7	54.5	0.59
Moderate/poor	35.3	45.5	

**Figure 4:**
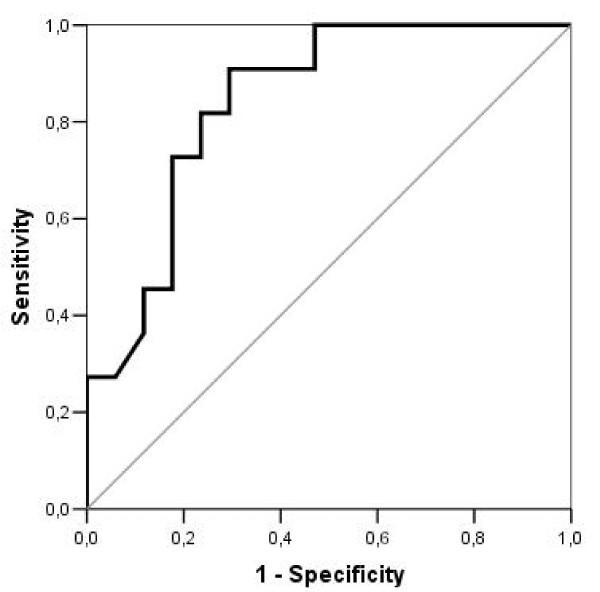
**Diagram of ROC curves for patients who received long course of radiotherapy**. Area 84.2%; p = 0.003.

The ROC test was made to discover the MVD cut-off which displayed a high risk of mortality after long RTX (Figure 4). The cut-off is 130 microvessel/mm^2^. We obtained 90.9% sensitivity and 70.6% specificity of MVD when the cut-off is 130.

**Figure 5:**
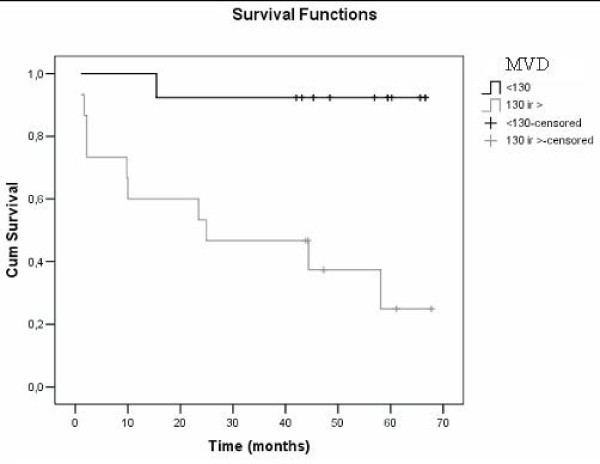
**Overall survival in subjects with a cut-off <130 versus cut-off >130**. Log rank test: p = 0.001.

According to the Kaplan-Meier method and as compared by the Mantel (log rank) test, the survival period tended to be longer when the tumor T was lower, there were no lymph node metastases, the tumor differentiation grade was better and the patient was female. The survival curve also showed a statistically-significant relationship between microvessel density and the survival period. When the MVD was lower than a cut off of 130, the survival period significantly increased (p = 0.001) (Figure 5). According to the Cox logistic proportional regression model, the mortality rate is significantly higher if the MVD is higher than 130 (vessel/mm^2^) (1953.047; p = 0.002), if the histological grade is moderate/poor (127.407; p = 0.013), if the tumor is T3/T4 (111.618; p = 0.014), and if the patient is male (17.92; p = 0.034) adjusted by other variable in model. (Table 4).

**Table 4:** Multivariate Cox proportional hazard analysis for mortality

	Relative risk (B)	95% CI for Exp(B)	p
MVD (130 ir >)	1953.047	14.816–257444.0	0.002

Age	0.96	0.864–1.066	0.446

Gender	17.92	1.243–258.432	0.034

Tumor	111.618	2.578–4833.392	0.014

Lymph node metastases	3.34	0.293–38.061	0.331

Histological grade	127.407	2.774–5852.496	0.013

## Discussion

Angiogenesis plays an important role in tumorogenesis and metastatic processes. There are few studies done that investigate the RTX influence on angiogenesis. Aside from direct tumor cytotoxic effects, RTX may also influence other physiologic parameters that are known to be important in colorectal cancer. In particular, little is known about the response of angiogenesis to this therapy. Increased type-IV collagenase activity (MMP-2 and MMP-9) following preoperative RTX in rectal cancer was recently discovered [11]. It is of note that these MMP participate in angiogenesis [12]. Increased MMP-2 activity was also reported in colon carcinoma and in osteosarcoma cell lines after 5 Gy of radiation at short time periods of up to 24 h. [13]. This evidence shows that RTX may temporarily increase the invasive potency of the tumor that leads to micrometastases or split tumor cells at the time of surgery [14]. According to Baeten et al. [15] RTX inhibits proliferation of EC despite a high percentage of residual EC in tumor tissue after prolonged RTX. This suggests a higher sensitivity of non-endothelial cells to RTX leading to a greater percentage of loss of these cells. MVD is one of the parameters that assesses the outcome of all the processes. Our results demonstrate that long RTX pre-treatment significantly decreased MVD in tumor tissue. Treatment using short RTX did not change the mean MVD as compared to tumor samples taken from those receiving no RTX; the only feature noticeable was that the dispersion of MVD was greater in a short RTX group as compared to no RTX group. It is of note that tumor samples from the short RTX group were taken one week after the radiation treatment. This suggests that the changes in the tumor tissue may be subsequent to the cytotoxic effects of RTX that lead to inflammation that begins in the tumor straight after RTX [16]. It was demonstrated that RTX up-regulated expression of adhesion molecules on endothelial cells, and as a result stimulated infiltration of leukocytes into tumor tissue. The increase in expression of EC adhesion molecules and of leukocyte infiltration after RTX suggests the activation of a local antitumor reaction [15]. The tumor samples from the patients that received a long course of RTX were taken 6 – 8 weeks after the RTX. During this period, cytotoxic effects and the inflammatory reaction induced by radiation treatment in the tumor tissue is diminishing, radiation induced necrotic lesions of the tumor are repaired by fibrous connective tissue [17]. We might speculate that neoplastic cells have less opportunity to invade surrounding tissue during surgery when MVD was decreased. Decreased invasion of neoplastic cells into surrounding tissues leads to a diminished local rate of recurrence of the tumor after preoperative RTX. In spite of this, the overall survival of the patients assigned to radiotherapy and surgery or assigned to surgery alone is similar [4]. This phenomenon can be explained by the development of distal metastases which might have spread at the beginning of RTX when expression of MMP and other angiogenic factors are increased [18,19].

In our study, we had limitation in sample size. We had enrolled all patients underwent surgery for rectal carcinoma in our surgical department during four years period. One third of patients underwent emergency operations or had tumors of another localisation (exclusion criteria). Another part had incomplete clinical data or unclear outcome of disease due to missing from follow-up. Some part of the patients was excluded because of the difficulties preparing the high quality slides from paraffin-embedded specimens. These reasons probably could have limited the reveal of statistically significant relationship between MVD and other prognostic markers such as tumor T, N, G. So our findings disagree with the findings of Gallego et al. They proved a relationship between MVD, G, N and the prognosis for survival [20,21]. Such a difference may be explained by the impact of the RTX on tumor angiogenesis. We demonstrated that there was a significant difference in MVD between patients who survived and those who did not survive during follow-up after combined treatment of a long course RTX and surgery. Thus MVD and the overall survival relationship discovered in our study indicates perhaps that a high MVD after a long course of RTX may show tumor resistance to radiotherapy in those tumor samples taken from patients who did not survive in comparison to those who did survive. This might indicate tumor resistance to RTX in a subset of tumors. According to Shia et al., recurrence-free survival time is better in patients with a fibrotic-type of stromal response with minimal inflammatory infiltrates [22]. MVD and the overall survival relationship discovered in our study indicates that high MVD after long RTX may show a high tumor resistance to radiotherapy.

## Conclusion

Our study using MVD in long course RTX reveals a very defined cut off and appears to have great prognostic potential. Based on the MVD changes, tumor response to RTX and individualized treatment could be evaluated. Further investigations are needed to uncover any changes in angiogenesis and in the inflammatory reaction during radiotherapy.

## Competing interests

The authors declare that they have no competing interests.

## Authors' contributions

SS conceived of the study, abstracted data, participated in assessement of histological fields, drafted and revised the manuscript. VL carried out the immunoassays, assessed histological fields, participated in the design of the study and helped to draft and approved the final manuscript. DP and AT participated in the planning and the design of the study, and in the analysis and discussion of the results. IN performed the statistical analysis. DPr carried out histopathological analyses and established pathological diagnosis. All authors read and approved the final manuscript.

## Pre-publication history

The pre-publication history for this paper can be accessed here:

http://www.biomedcentral.com/1471-2407/9/95/prepub
